# Art and Disease

**Published:** 1894-03-10

**Authors:** 


### Art and Disease.

### VII.—ASPECTS OF DEATH.

Greek art recognises death under its noblest aspects alone. The perfection of repose, the completion of conflict, the stilling of all disquiet and emotion form the motive of the frequent representations of the dead to be found on bas reliefs and Greek vases. M. Charcot observes that in nearly all these examples the death has been a violent and sudden one. The wound is often visible, but the body of the slain retains every mark of vigour and beauty, and betrays that the spirit has passed away only by the inertia of the muscles. The motionless limbs have settled in obedience to the laws of gravity, but with all the flaccidity of death, they have in no degree lost the harmonious proportions of life. Two famous representations of the dying in ancient art—the Dying Gladiator and the Laocoon—will occur to every one. The repressed force of the former, in which the death struggle is indicated with unshrinking fidelity, endues it with dignity, and banishes every trace of the repulsive. But the Laocoon rises to a higher level, and shows the hero supreme in the hour of mortal agony—balancing, to use the words of Winckelmann. by the grandeur of his soul, the sufferings of his body,

Christian art, represented at its earliest in the frescoes of the catacombs, has no pictures of death. Neither the sufferings of the Martyrs, nor the Passion and Death of Christ, formed part in the subjects of these early artists. It is surprising to learn that the Figure of the Crucifix is of comparatively late origin. The Cross was a symbol in use from earliest times, but the Figure of the Christ was according to Emeric David,* not carved upon it, until the beginning of the eighth century, when John VII., a Greek by birth, was elected Pope, and set up a crucifix in the chapel of St. Peter. The Saviour is robed in a long tunic down to the feet, and no token of suffering or death is visible in the face. For some centuries onward every trace of emaciation or present agony was rigidly excluded from all representations of the Crucifixion. But in the thirteenth and fourteenth centuries artists sought to stimulate the devotions of the faithful by more realistic studies, and the effect is all the more painful, from the fact that the symptoms of anguish are seldom even true to life. It would be impossible to recall the numerous beautiful conceptions of the Crucifixion dating from the times of the Renaissance. In the greater proportion of these the moment chosen by the artist is that preceding death, and it is in the Descent from the Cross, or the still more popular Pieta, which represents the Virgin mourning over the body of her Son, that the figure of the dead Christ is most vividly presented. M. Charcot points out two distinct phases in this connection.† "Sometimes the dead Christ is represented when muscular rigidity has extended to all the limbs. The stiffened corpse maintains itself in a fixed position, as Van der Veyden shows us in two examples of a descent from the Cross, one at Vienna, and the other at the Louvre, full of fine observation. On the other hand, Rembrandt shows the body of the Lord in a state of the most complete resolution. It is difficult to indicate with more vigour and air of reality that state of absolute flaccidity of the body, in which the muscles have lost all their tonicity, than he has succeeded in doing in his Descent from the Cross at the Munich Museum." Every description of torture and painful death was fluently delineated by the mediæval artists in depicting the history of the saints. In many quaint and not unpleasing pictures of martyrdoms the principal sufferer is absolutely unconcerned by the ravages committed on his body, and reveals a state of pure beatitude or angelic triumph in the midst of his torments. In other such representations a too successful realism renders the effect altogether terrifying. We quote a passage from Ruskin's comments on "The Scuola di San Rocco" in illustration of a San Sebastian, by Tintoretto, which has successfully avoided both these errors.‡ "There is no effort at any expression of angelic or saintly resignation; the effort is simply to realise the fact of the martyrdom, and it seems to me that this is done to an extent not even attempted by any other painter. I never saw a man die a violent death, and therefore cannot say whether this figure be true or not, but it gives the grandest and most intense impression of truth. The figure is dead, and well it may be, for there is one arrow through the forehead and another through the heart; but the eyes are open though glazed, and the body is rigid in the position in which it last stood, the left arm raised and the left limb advanced, while the dead eyes are still fixed in the direction from which the arrows came; but the most characteristic feature is the way these arrows are fixed. In the common martyrdoms of St. Sebastian they are stuck into him here and there like pins, as if they had been shot from a great distance and come faltering down, entering the flesh but a little way and rather bleeding the saint to death than mortally wounding him, but Tintoretto had no such ideas about archery. All the arrows in the saint's body lie straight in the same direction, broad feathered and strong shafted, and sent apparently with the force of thunderbolts. The face, in spite of its ghastliness, is beautiful and has been serene, and the light which enters first and glistens on the plumes of the arrows, dies softly away upon the curling hair and mixes with the glory upon the forehead."

It is, as might be expected, among funeral monuments that the most faithful studies of death are to be found. The nobler monuments follow Greek models and represent their dead as lying wrapped in slumber. Chantrey's Monument of the Sleeping Children in Lichfield Cathedral is perhaps the most beautiful English example of this conception. But in Renaissance times when sober reticence came to be regarded as timidity, and artists learnt to claim all nature for their inheritance, the very charnel house was forced to give up its secrets, and the most repulsive features of decay and putrefaction were portrayed with stern exactness. The subject of the monument was shown in full life and vigour above, while below the decaying corpse formed a frightful contrast. The tombs of Westminster Abbey show more than one example of this morbid or theatrical phase, over which there is no temptation to linger.

* Histoire de la Peniture au Moyen Age, par Emeric David.

† Les Diffromes et les Malades dans l'Art, par J. M. Charcot et Paul Richer, page 129.

‡ Stones of Venice, by John Ruskin. Fourth ed.; vol. 3, page 333.

